# Effects of Top-Pruning Intensity Gradient on Root System Architecture and Allometric Patterns in *Pinus yunnanensis* Franch. Seedlings

**DOI:** 10.3390/plants14203210

**Published:** 2025-10-19

**Authors:** Guangpeng Tang, Jianzhen Liao, Yulan Xu, Nianhui Cai

**Affiliations:** 1 Key Laboratory of Forest Resources Conservation and Utilization in the Southwest Mountains of China, Ministry of Education, Southwest Forestry University, Kunming 650224, China; tangguangpeng@swfu.edu.cn (G.T.); liaojianzhen@swfu.edu.cn (J.L.); xuyulan@swfu.edu.cn (Y.X.); 2 Key Laboratory of National Forestry and Grassland Administration on Biodiversity Conservation in Southwest China, Southwest Forestry University, Kunming 650224, China

**Keywords:** biomass accumulation and allocation, root changes, growth characteristics, *Pinus yunnanensis* Franch. seedlings

## Abstract

*Pinus yunnanensis*, is an ecologically and economically important tree species in southwestern China. However, its natural renewal is relatively lagging behind, and it is difficult to achieve sustainable development. Apical removal (top-pruning) can eliminate apical dominance, stimulate sprouting, and provide high-quality scions for clonal propagation. Root systems are a critical foundation for sprouting capacity. In this study, one-year-old *P. yunnanensis* seedlings were subjected to four treatments: removal of 3/4 (H1), 2/4 (H2), or 1/4 (H3) of the seedling height, and a non-topped control group (CK). The objective was to investigate the seedlings’ responses in terms of root morphology, biomass allocation, and allometric growth. The results showed that by May, biomass allocation in the topped treatments increased by 13.37%, 11.01%, and 7.86%, respectively, compared with the control, and also exhibited higher coefficients of variation. Under the H2 treatment, both fine and coarse roots accounted for a higher proportion of total root biomass and displayed stronger water-retention stability. With increased top-pruning intensity and time, root volume, specific root length, root tissue density, and root tip number were the first to respond, indicating the onset of allometric growth. Notably, in May, the growth rate of specific root surface area followed the order: H3 > H1 > CK > H2. These findings suggest that the root system adapts to environmental changes by modulating growth patterns among various indicators to optimize resource allocation and enhance adaptability.

## 1. Introduction

*P. yunnanensis* Franch., commonly known as Yunnan pine, is an endemic species in southwestern China. It is primarily distributed in the western regions at altitudes ranging from 1500 to 3000 m [1] and serves as a pioneer species in the natural regeneration of barren hills in the Yunnan–Guizhou Plateau. This species holds significant ecological and economic value [2], contributing to soil and water conservation, windbreaks, and sand fixation. Additionally, it is utilized in resin production, papermaking, tannin extraction, and medicine [3]. Its pollen is rich in various amino acids, vitamins, trace elements, and enzymes, which help enhance cellular activity and promote metabolism. However, in recent years, the natural regeneration of *P. yunnanensis* has lagged behind, diminishing its ecological contribution to environmental restoration. Addressing this issue through appropriate interventions is essential for promoting green and sustainable development. Apical removal (top-pruning) has been shown to eliminate apical dominance [4] and stimulate sprouting capacity in seedlings [5], thereby laying the foundation for the development of high-quality scions. These scions can be used in asexual propagation methods such as cutting [6] and grafting [7], supporting the ecological restoration and expansion of *P. yunnanensis* through clonal propagation [8]. As the primary organ responsible for nutrient absorption and transport, the root system plays a pivotal role in sprout development following apical removal. Thus, this study focuses on the root responses of *P. yunnanensis* seedlings under varying intensities of top-pruning stress, aiming to provide a scientific basis for the differentiation and formation of high-quality scions.

Roots are essential components of plant architecture [9], playing a critical role in supporting normal growth and development [10]. Their spatial distribution, density, and morphology [11,12,13], are major factors affecting water and mineral absorption [14]. Based on root diameter, the root system is typically classified into fine roots (≤2 mm) and coarse roots (>2 mm) [15,16]. Fine roots are the most vital components for nutrient uptake, anchorage, and support of the above-ground parts. They also consume photosynthates through respiration and turnover while contributing organic matter to the soil [17]. As such, fine roots act not only as a significant “sink” in forest ecosystems but also as key “sources” of nutrients and soil carbon, making them crucial to understanding material cycling and energy flow in forest ecosystems [18,19]. The number, morphology, distribution, and configuration of fine roots influence nutrient and water uptake, are closely related to root longevity and turnover, and ultimately determine a plant’s strategy for acquiring soil resources. These characteristics have profound implications for forest carbon balance and nutrient cycling [20]. In contrast, the primary physiological roles of coarse roots are to provide structural support for fine root growth and to store photosynthetic products [21].

In plants, biomass accumulation and allocation represent both the integrated outcome of net carbon acquisition and a critical driving factor for plant growth [22]. Numerous traits related to biomass allocation, metabolic rate, and nutrient stoichiometry vary along the global spectrum of plant size diversity [17,23,24,25,26]. Among these, the theory of allometric growth in biomass allocation [27] posits that resource allocation patterns among different organs shift systematically with plant size and are largely independent of local environmental conditions [28,29]. Allometric growth refers to the disproportionate scaling between two traits of an organism [30]. Studies have shown that when plants are subjected to damage or stress, they tend to allocate more resources to organs responsible for acquiring limiting resources, thereby maintaining optimal growth rates under adverse conditions [31]. As a fundamental framework for describing resource distribution, allometric relationships serve as important parameters for predicting plant growth and vegetation productivity [32]. Moreover, allometric adjustment is likely a critical component of both short- and long-term plant responses to abiotic conditions [33].

## 2. Results

### 2.1. Effects of Different Top-Pruning Intensities on Root Biomass Accumulation, Allocation, and Relative Water Content

**Figure 1:**
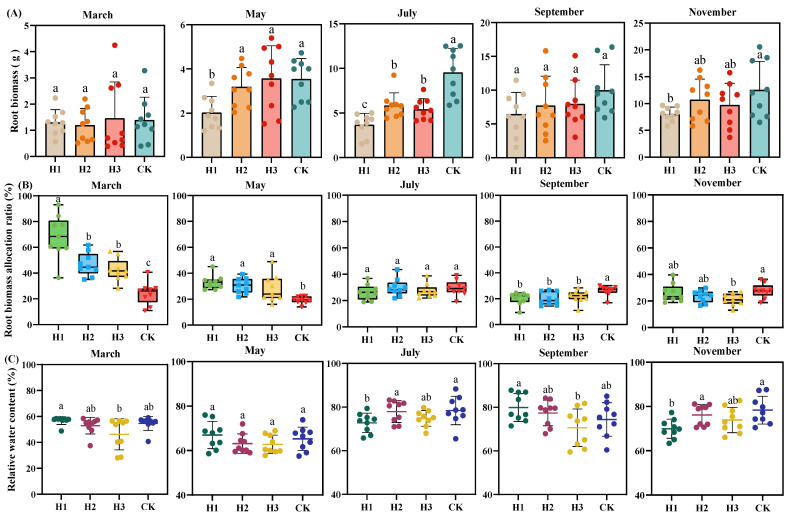
Effects of different top-pruning intensities on root biomass accumulation (**A**), allocation (**B**), and relative water content (**C**) of *P. yunnanensis* seedlings. (Note: Different lowercase letters in the figure indicate significant differences between treatments. ANOVA followed by *t*-test, n = 9, *p* < 0.05. Error bars represent the mean ± standard error of the mean (SEM)).

As shown in Figure 1A, the root biomass accumulation of seedlings followed the trend CK > H2 > H3 > H1. During the growth process, a significant difference (*p* < 0.05) was observed only between H1 and CK, while differences among other treatments were not significant. Figure 1B indicates that root biomass allocation per individual initially decreased and then increased. In May, the biomass allocation under top-pruning treatments (H1, H2, H3) was significantly higher than that of CK by 13.37%, 11.01%, and 7.86%, respectively (*p* < 0.05). However, by September, this trend reversed, and the biomass allocation of CK significantly increased by 4.58% to 6.12%. Figure 1C shows that in March, right after top-pruning, the relative water content (RWC) of roots was low. Among treatments, H1 had the highest RWC (56.79%) and H3 the lowest (46.24%), with a significant difference between the two (*p* < 0.05). H2 maintained a stable RWC over time, indicating good water retention and drought resistance. Notably, H1 and H3 displayed opposite trends in RWC: when RWC in H1 increased, H3 decreased, and vice versa.

### 2.2. Effects of Different Top-Pruning Intensities on Root Diameter Distribution

**Figure 2:**
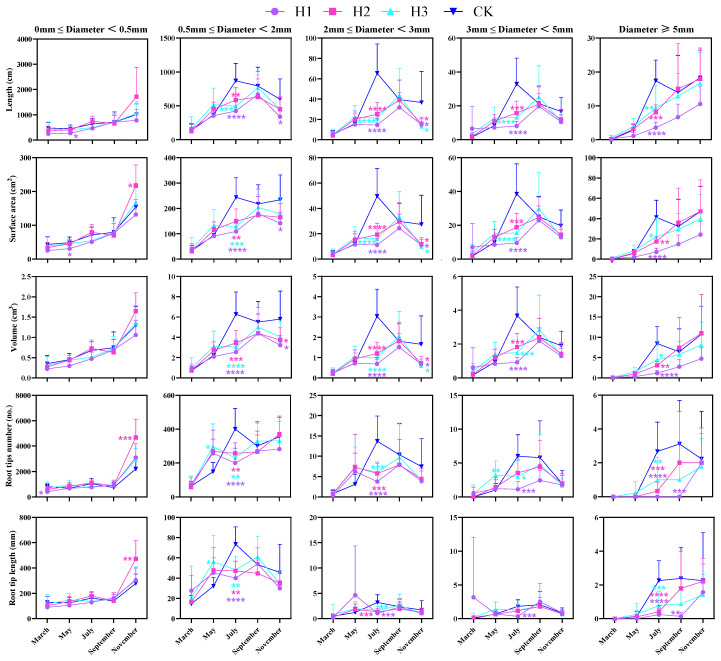
Changes in seedling root diameter class under different top-pruning intensity (Note: * Indicates significant differences between pruning treatments (H1, H2, H3) and the control (CK). ANOVA + *t*-test, n = 9, * *p* < 0.05, ** *p* < 0.01, *** *p* < 0.001, **** *p* < 0.0001. Error bars represent the mean ± standard error of the mean (SEM)).

Analysis of root diameter under four treatments showed that roots with diameters of 0–0.5 mm and 0.5–2 mm accounted for the majority of total root length, followed by roots of 2–3 mm and 3–5 mm, while roots over 5 mm were least common (Figure 2). This indicates that *P. yunnanensis* seedlings primarily develop fine roots (0–2 mm), which are crucial for nutrient absorption and growth. Further analysis revealed that, except for roots of 0–0.5 mm diameter, root length increments in July were significantly greater in CK than in topped treatments (*p* < 0.01). Overall, root diameter performance followed the pattern: CK > H3 > H2 > H1.

### 2.3. Effects of Different Top-Pruning Intensities on Fine and Coarse Roots

**Figure 3:**
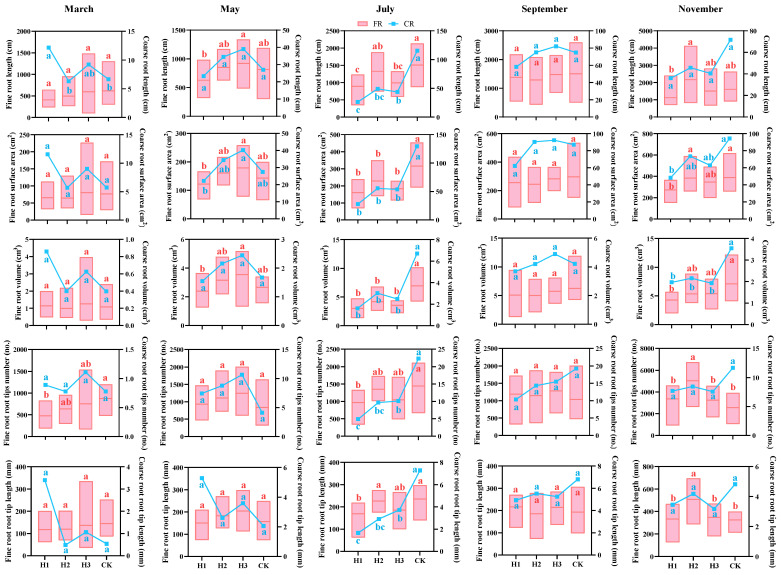
Difference analysis of each index of fine roots and coarse roots of seedlings under different topping intensities (Note: Different lowercase letters represent significant differences between treatments. ANOVA + *t*-test, n = 9, *p* < 0.05.).

Based on Brunnre et al. [15] and Wang et al. [16] roots with diameters ≤ 2 mm were classified as fine roots, and those > 2 mm as coarse roots. In March (Figure 3), fine root parameters were highest under H3 and lowest under H1, whereas coarse root development showed the opposite trend (H1 > H2 ≈ CK). This suggests that increased top-pruning intensity shifted root development strategies toward coarse roots to enhance nutrient uptake under stress. By May, both fine and coarse roots peaked under H3, showing significant differences (*p* < 0.05) in total length, surface area, and volume. In July, CK reached the highest values in all root indicators, significantly outperforming topped treatments, aligning with Figure 2 results and indicating a temporal shift in root development due to top-pruning.

In September, H3 showed advantages in coarse roots, with increases of 10.0%, 6.1%, and 16.2% in total root length, surface area, and volume, respectively, over CK. Root tip number and length were also enhanced by 24% and 10.9%. In November, H2 significantly outperformed CK in fine root tip number and length by 56.5% and 97.4%, respectively. However, CK still exhibited superior performance in coarse root total length and surface area (*p* < 0.05).

### 2.4. Correlation Analysis of Root Morphological Traits

**Figure 4:**
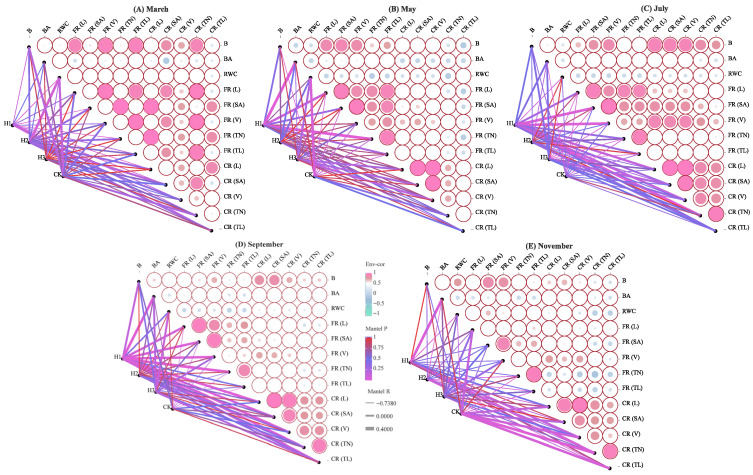
Correlation analysis of root morphology indexes of *P. yunnanensis* seedlings (Note: B represents Biomass; RWC represents Relative Water Content; BA represents biomass allocation; FR represents Fine Root; CR represents Coarse Root; the size of the circles represents the strength of the significance, with larger circles indicating stronger significance and smaller circles indicating weaker significance).

Time-series analysis of root traits and biomass accumulation (Figure 4) revealed significant synergistic patterns (*p* < 0.01) with distinct stage-specific responses. In March (Figure 4A), coarse and fine roots developed in coordination. Coarse root length (CR-L) was highly positively correlated with fine root length (FR-L), volume (FR-V), and tip length (FR-TL) (*R* = 0.377~0.498, *p* < 0.01), suggesting H2 activated fine root plasticity to promote coarse root growth. H2 also correlated positively with relative water content, indicating improved water status. In May (Figure 4B), root biomass was mainly influenced by fine root traits (FR-L/V/TL), while CR-L was significantly correlated with CR-SA and CR-V (*p* < 0.05), demonstrating functional division between fine root absorption and coarse root transport. In July (Figure 4C), correlations strengthened; biomass (B) was significantly linked with both FR and CR, especially CR. As top-pruning intensity decreased, FR and CR indices aligned more closely. In September and November (Figure 4D,E), root activity declined as seedlings entered a slower growth phase, reducing inter-index correlations. In mid-September, higher top-pruning intensity promoted biomass (BA) and root development (FR, CR). By November, CK facilitated greater coarse root growth.

### 2.5. Variation Analysis of Root Morphological Traits Under Different Top-Pruning Intensities

**Figure 5:**
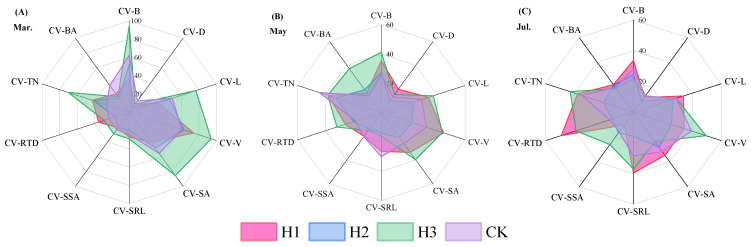

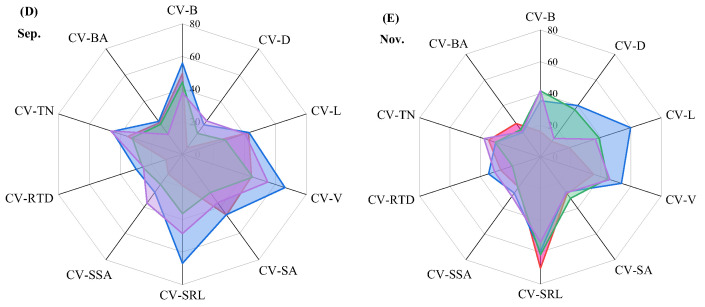
Coefficient of variation in root morphological characteristics of *P. yunnanensis* seedlings under different top-pruning intensities. (Note: B represents Biomass; BA represents Biomass Allocation; TN represents Root Tips Number; RTD represents Root Tissue Density; SSA represents Specific Surface Arez; SRL represents Specific root Length; SA represents Surface Area; V represents Volume; L represents Length; D represents Diameter; Same below).

The coefficient of variation (CV) was calculated as CV = standard deviation/mean × 100%. CV values of root traits ranged from 5.96% to 94.76%, averaging 32.45% (Figure 5). The ranking of CV by trait was: Volume > Biomass > SSA > Length > Tip Number > RTD > SRL > Biomass Allocation > SSA > Diameter. Across treatments, CVs ranged as follows: H1 (4.74~73.88%), H2 (5.96~66.90%), H3 (10.82~94.76%), and CK (13.08~62.23%), with the order H3 > H1 > H2 > CK. Overall, top-pruning increased trait variability, and CVs showed a trend of rising then falling with increasing top-pruning intensity.

### 2.6. Allometric Growth Relationships of Root Traits Under Different Top-Pruning Intensities

**Figure 6:**
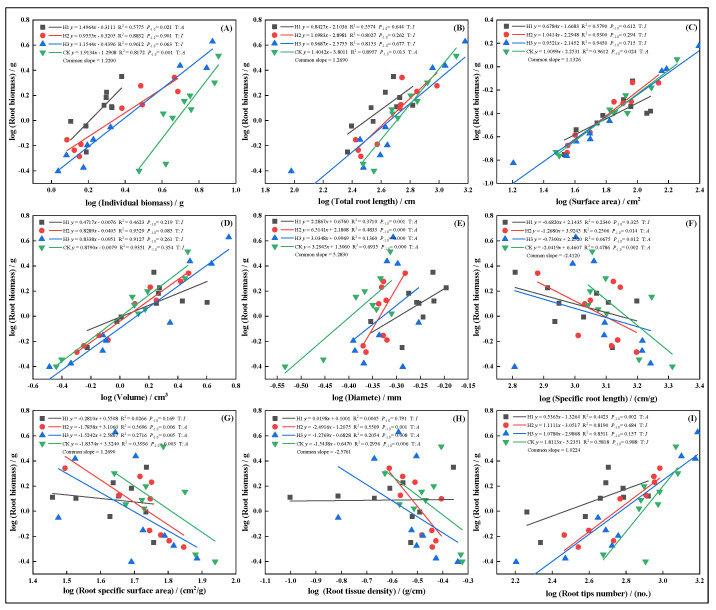
In March, the allometric relationship between root biomass and biomass individual plant (**A**), total root length (**B**), root surface area (**C**), root volume (**D**), root diameter (**E**), root specific length (**F**), root specific surface area (**G**), root tissue density (**H**), root tip number (**I**).

**Figure 7:**
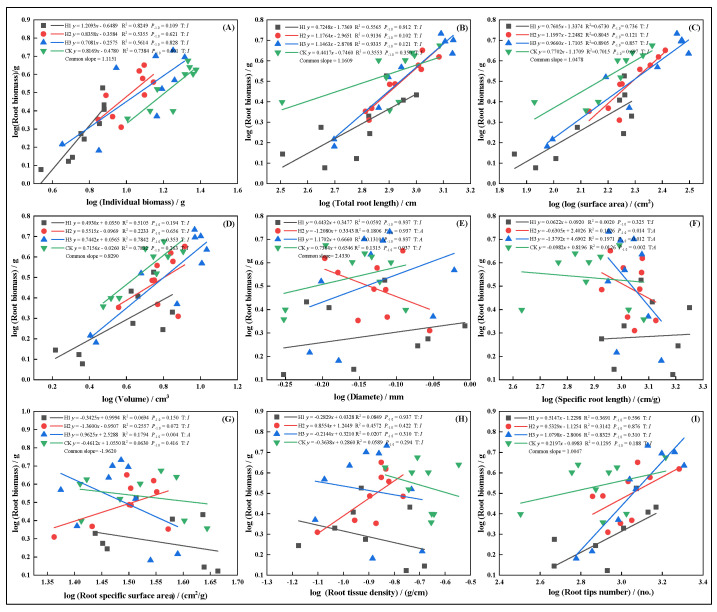
In May, the allometric relationship between root biomass and biomass individual plant (**A**), total root length (**B**), root surface area (**C**), root volume (**D**), root diameter (**E**), root specific length (**F**), root specific surface area (**G**), root tissue density (**H**), root tip number (**I**).

In March, increasing top-pruning intensity altered the allometric trajectories between root biomass and individual plant biomass, total root length, surface area, volume, diameter, specific root length, specific surface area, tissue density, and root tip number (Figure 6. All traits, except root volume (Figure 6D) and root tip number (Figure 6I), exhibited allometric growth under CK. In H1, individual biomass (Figure 6A) and tissue density (Figure 7I) showed faster growth, indicating allometric scaling. However, SRL (Figure 6F), SSA (Figure 6G), and RTD (Figure 6H) in H1 exhibited isometric relationships.

**Figure 8:**
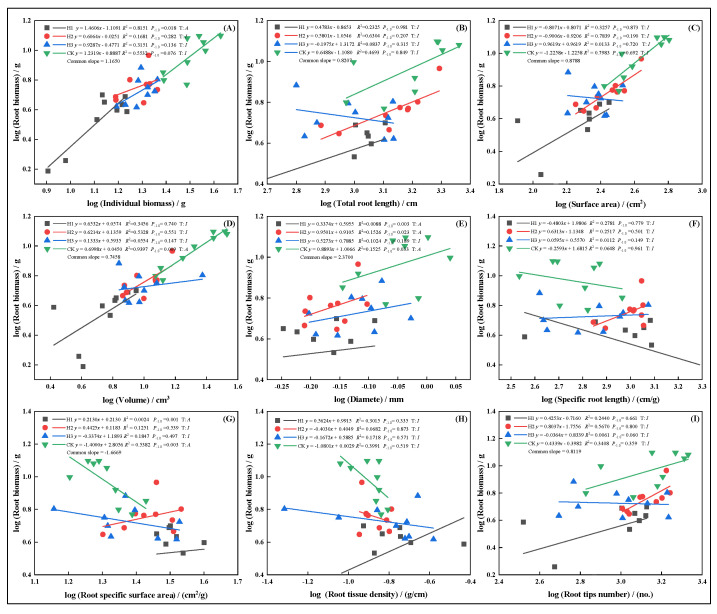
In July, the allometric relationship between root biomass and biomass individual plant (**A**), total root length (**B**), root surface area (**C**), root volume (**D**), root diameter (**E**), root specific length (**F**), root specific surface area (**G**), root tissue density (**H**), root tip number (**I**).

In May, growth relationships between root biomass and other traits were more stable, generally showing isometric growth. However, SSA under H3 displayed an allometric pattern (Figure 7G), with the growth rate ranking: H3 > H1 > CK > H2. Root diameter showed the opposite trend. In July, topped groups (H1, H2, H3) displayed isometric growth in root volume (Figure 8D), suggesting top-pruning suppressed root volume expansion. Root biomass maintained proportional relationships with total root length (Figure 8B), surface area (Figure 8C), and SRL (Figure 8F).

**Figure 9:**
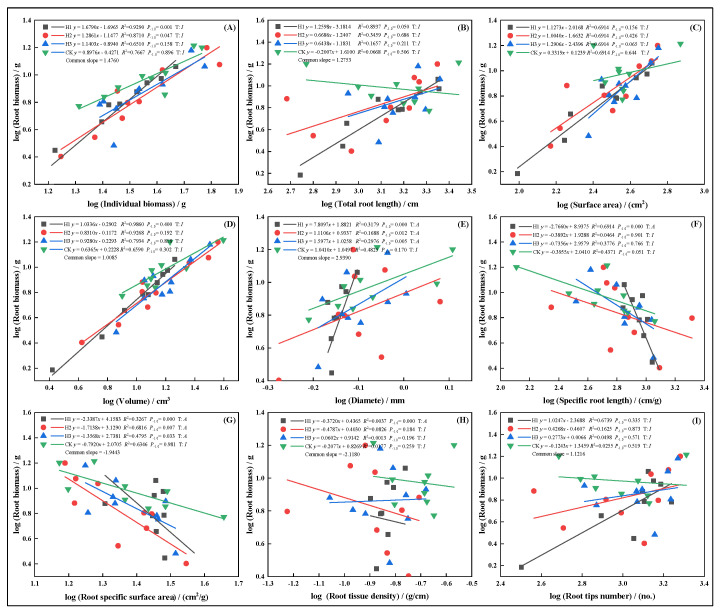
In September, the allometric relationship between root biomass and biomass individual plant (**A**), total root length (**B**), root surface area (**C**), root volume (**D**), root diameter (**E**), root specific length (**F**), root specific surface area (**G**), root tissue density (**H**), root tip number (**I**).

**Figure 10:**
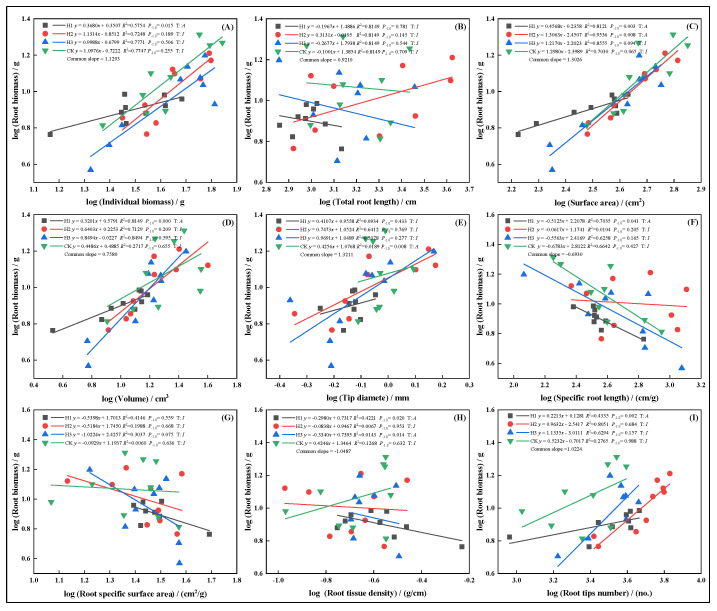
In November, the allometric relationship between root biomass and biomass individual plant (**A**), total root length (**B**), root surface area (**C**), root volume (**D**), root diameter (**E**), root specific length (**F**), root specific surface area (**G**), root tissue density (**H**), root tip number (**I**).

In September, root biomass and diameter (Figure 9E), as well as SSA (Figure 9G), exhibited allometric growth under top-pruning, indicating promoted expansion of root diameter and surface area. Growth relationships followed: H3 > H1 > H2 > CK (Figure 9C). In November, under H1, allometric growth was observed in individual biomass (Figure 10A), root surface area (Figure 10C), volume (Figure 10D), SRL (Figure 10F), RTD (Figure 10H), and root tip number (Figure 10I), while other treatments showed stable isometric growth.

## 3. Discussion

### 3.1. Response of Root Morphological Traits to Different Top-Pruning Intensities

Root morphology plays a crucial role in maintaining the balance of resource allocation and utilization during root growth and activity [34]. In this study, top-pruning treatments promoted root stress responses and correspondingly increased the coefficient of variation, enhancing the adaptability of root growth. These physiological traits exhibited significant differences. In July, the increment of fine roots was notably higher, a result consistent with Malhotra et al. [35] study on shrub fine roots increasing with temperature during the growing season, indicating the key role of fine roots in the growth of *P. yunnanensis* seedlings (Figure 3). The correlation between root diameter and biomass varied. Biomass showed a positive correlation with roots in the 0–2 mm and 2–3 mm diameter ranges, as the increase in fine and coarse roots enhanced the root system’s ability to utilize water and nutrients [36], thus promoting root growth and increasing biomass. Root biomass allocation is vital for tree anchorage, moisture and nutrient absorption, and soil improvement [37,38]. Biomass accumulation in the root system of *P. yunnanensis* seedlings followed the trend: CK > H2 > H3 > H1, with corresponding differences in root biomass allocation and relative water content. This pattern is similar to the responses of other plants under environmental stress; for example, under drought stress, some plants increase root biomass allocation and improve relative water content to enhance water absorption and retention capacity [9]. In this study, top-pruning treatment, as a form of artificial stress, altered the physiological traits of the seedlings, with the H2 treatment demonstrating better water retention and drought resistance. This may serve as an effective mechanism for coping with top-pruning stress. Since the function and metabolic activities of plant organs constrain nutrient allocation between them [39], and biomass allocation patterns are fundamental for estimating primary productivity and ecosystem process rates [33], understanding root biomass allocation and morphological changes is crucial for evaluating forest functions and biogeochemical cycles. Moreover, the diverse responses of plants to environmental stress reflect evolutionary adaptation strategies that ensure growth and survival under varying conditions. The study further suggests that moderate top-pruning can enhance root systems’ efficiency in acquiring and utilizing resources, representing an adaptive strategy for maintaining overall growth and development under top-pruning stress.

### 3.2. Response of Seedling Allometric Growth to Different Top-Pruning Intensities

Compared with the untreated control, top-pruning treatments promoted earlier changes in root diameter in *P. yunnanensis* seedlings (Figure 9 and Figure 10). This indicates that seedlings can quickly respond to top-pruning stress, thereby promoting underground growth to better absorb nutrients and water to cope with environmental changes. Similar findings have been observed in pruning experiments with other trees, where root systems adjust their growth and morphology to accommodate changes in the above-ground parts [5]. This is due to the close connection between above-ground and below-ground parts, where changes in the former affect the growth and development of the latter, and such adaptive changes in the root system help maintain the overall growth balance of the plant. Broadly speaking, allometric growth relationships are defined as the covariance between an organism’s biomass and its morphological and physiological traits [23]. The differences in allometric growth indices between different traits can indicate nutrient allocation patterns among different plant organs [40]. In this study, the analysis of allometric growth relationships between root biomass and individual biomass showed that top-pruning accelerated root biomass accumulation in seedlings, with above-ground disturbances stimulating root growth, thereby promoting overall plant development [41]. Significant differences were found in allometric growth indices of *P. yunnanensis* seedlings under different top-pruning intensities. Under the H1 treatment, the relationship between root biomass and root volume exhibited isometric growth, indicating that the top-pruning treatment inhibited the increase in root volume to some extent. This aligns with the findings of Warton et al. [42], who stated that allometric growth relationships reflect plant adaptation strategies to environmental changes. The H3 treatment, on the other hand, showed a higher growth rate in allometric growth, possibly due to the redistribution of resources in the root system after top-pruning. As noted by Rudgers et al. [33], allometric growth modulation is an essential part of plant responses to abiotic conditions, whether short- or long-term, and the allometric growth patterns of *P. yunnanensis* seedlings under different top-pruning intensities represent adaptive adjustments to top-pruning stress.

Plants exhibit considerable variation in size and morphology [43], and one major consequence of changes in organism size is the alteration of the surface area-to-volume ratio, which significantly impacts the utilization of biological resources, growth, development, reproduction, and survival [44,45,46]. In this experiment, following top-pruning stress, root biomass, root surface area, and root volume of *P. yunnanensis* seedlings exhibited allometric growth relationships over time (Figure 10C,D). This suggests that the seedlings respond to external stress by altering metabolic pathways, redistributing energy, and adjusting root morphology, further supporting this viewpoint. Plants optimize resource allocation by adjusting allometric growth relationships in response to various environmental changes and internal growth demands, thereby achieving better growth and development. The allometric growth response of *P. yunnanensis* seedlings to top-pruning stress is a critical mechanism for adapting to environmental changes, reflecting the diversity and complexity of plant adaptive strategies.

## 4. Materials and Methods

### 4.1. Study Area

**Figure 11:**
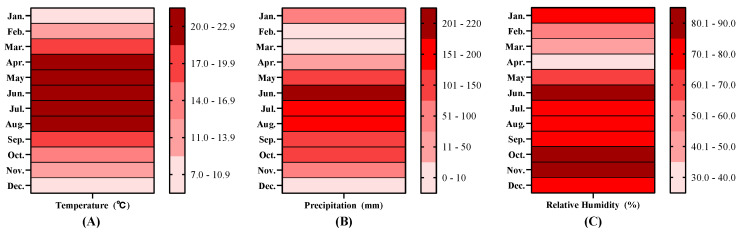
The temperature (**A**), relative humidity (**B**) and precipitation (**C**) of the study area in that year. (Note: Data are based on historical reanalysis datasets from the European Centre for Medium-Range Weather Forecasts (ECMWF)/National Aeronautics and Space Administration (NASA: Washington, DC, USA), provided by www.xihe-energy.com accessed on 15 October 2025 [47].

The experiment was conducted in the Greenhouse Complex of Southwest Forestry University (25°04′00″ N, 102°45′41″ E). This site is characterized by a northern subtropical semi-humid plateau monsoon climate with distinct dry and wet seasons, at an elevation of approximately 1945 m. The annual average sunshine duration is 2445.6 h, with a sunshine percentage of 56%. The annual total solar radiation is 543.36 kJ/cm^2^, and the mean annual evaporation is 1856.4 mm. In the year of the study, the average air temperature was 17.1 °C, with the highest monthly average of 21.7 °C in July and the lowest of 9.6 °C in January (Figure 11A). The average annual relative humidity was 68.8%, with relatively low humidity in spring (Figure 11B). The annual precipitation was approximately 1100 mm, mainly concentrated between May and October, with peak monthly rainfall values of 219 mm (June), 180 mm (July), and 196 mm (August) (Figure 11C). The soil in the study area is acidic, low-phosphorus red soil with a pH of 6.0–6.2 and total phosphorus content ranging from 0.90 to 1.22 g/kg.

### 4.2. Experimental Design

Seeds of *P. yunnanensis* were collected from a clonal seed orchard in Midu County, Yunnan Province. The mother trees were healthy and vigorous, and mature cones harvested during the current year were used. After labeling and sun-drying, the seeds were disinfected and screened. The selected seeds were then sown in seedling trays for germination and subsequently hardened off. In mid-March 2023, healthy and uniformly growing seedlings were transplanted into nursery pots with a base diameter of 18 cm and a height of 32 cm. At the time of pruning (March 2024), the one-year-old *P. yunnanensis* seedlings used had an average height of 6.1 cm and a root collar diameter of approximately 4.51 mm. The potting substrate consisted of a 3:1 mixture of humus soil and red soil (acidic, low-phosphorus). Uniform seedlings were selected for subsequent experiments. All seedlings were managed under identical conditions, with weeding every two weeks and irrigation every 3 to 5 days to ensure full watering.

After one year of cultivation, local surveys were conducted on the seedlings. On 7 March 2024, seedlings with consistent growth performance were selected for apical removal treatments. The cut surfaces were sealed with paint to reduce water loss. Four treatments were established: H1 involved removing 3/4 of the seedling height, H2 involved removing 2/4, H3 involved removing 1/4, and CK served as the control with no top-pruning. Each treatment group consisted of 162 seedlings, totaling 648 seedlings.

### 4.3. Root Measurement

The first round of sampling was conducted at the end of March 2024, followed by subsequent samplings in May, July, September, and November. In each sampling period, three seedlings were randomly selected from each treatment group, with three replicates, yielding a total of 36 seedlings (4 treatments × 3 replicates × 3 seedlings). The entire root system was excavated using the full excavation method, then rinsed with clean water, drained, and packed in labeled bags for transport to the laboratory for analysis. Fresh root biomass was measured using an electronic balance (accuracy: 0.0001 g). Root biomass allocation ratio was calculated as:

Root biomass allocation (%) = (Root biomass/Individual biomass) × 100%.

Root morphological traits were assessed using an Epson root scanner, and parameters including total root length (cm), root surface area (cm^2^), average root diameter (mm), and total root volume (cm^3^) were obtained using the WinRHIZO 2021 analysis software. All measurements were recorded to four decimal places. According to reference [15,16], the roots were divided into coarse roots (diameter > 2 mm) and fine roots (diameter ≤ 2 mm), and the corresponding analysis was carried out.

RWC was measured in the method described by Boyer (1969) [48] with minor modifications. Roots were weighed immediately after harvest to determine the fresh mass (FM). The turgid mass (TM) was then recorded after the roots were transferred to de-ionized water and maintained in a dim environment for 4 h. Dry mass (DM) was measured after drying at 80 °C for 24 h. RWC was calculated using the following equation: RWC% = (FM − DM)/(TM − DM) × 100 [49].

### 4.4. Data Analysis

Data were organized using Microsoft Excel 2020. One-way analysis of variance (ANOVA) was performed on root biomass and relative water content using SPSS 20.0. Independent-sample *t*-tests were used to compare fine roots and coarse roots. All results are expressed as mean ± standard error (SE). Pearson and Spearman correlation analyses were conducted to assess relationships among different traits and treatments.

The coefficient of variation (CV) was calculated as CV = standard deviation/mean × 100%. Allometric growth relationships were described using the equation *y* = *ax*^*b*^ [50]), which can be linearized as log *y* = log *a* + *b* log *x*, where *y* represents root biomass and *x* refers to individual traits such as Individual Biomass, Total Root Length (L), Surface Area (SA), Volume (V), Root Diameter (D), Root Specific Surface Area (SSA), Specific Root Length (SRL), and Root Tissue Density (RTD). In this equation, a is the intercept indicating the trait baseline, and b is the allometric exponent (slope). When *b* = 1, growth is considered isometric; when *b* ≠ 1, it indicates allometric growth. Differences in slope between groups were compared, and if no significant difference was found, a common slope was reported. Parameter estimation for the allometric models was conducted using Standardized Major Axis (SMA) regression, implemented through the “Smatr” package in R [36,42]. Figures were produced using Origin 2021, GraphPad Prism 9.0, and the online platform https://www.omicshare.com accessed on 15 October 2025.

## 5. Conclusions

This study explored the effects of different top-pruning intensities on the root morphology, physiological traits, and allometric growth relationships of *P. yunnanensis* seedlings. The results show that top-pruning treatments significantly affect root biomass accumulation and allocation. The H2 and H3 treatments demonstrated superior growth recovery, with fine roots (0–2 mm) making up a significantly higher proportion compared to the control. Additionally, the H2 treatment exhibited more stable relative water content and stronger drought resistance. Seedlings enhanced their water and nutrient absorption efficiency by increasing the proportion of fine roots, while coarse roots (>2 mm) provided structural support for fine root expansion. Under top-pruning stress, the root system adjusted growth patterns between various traits to optimize resource allocation. The H3 treatment exhibited higher allometric growth rates for most indicators, indicating that moderate top-pruning can accelerate root functional differentiation, thereby enhancing the seedling’s adaptability to environmental changes.

## Data Availability

All data generated or analyzed during this study are included in this article. All data are available upon reasonable request.
